# Generalized framework for context-specific metabolic model extraction methods

**DOI:** 10.3389/fpls.2014.00491

**Published:** 2014-09-19

**Authors:** Semidán Robaina Estévez, Zoran Nikoloski

**Affiliations:** Systems Biology and Mathematical Modeling Group, Max Planck Institute of Molecular Plant PhysiologyPotsdam-Golm, Germany

**Keywords:** genome-scale models, high-throughput data, data integration, context-specific models, mathematical programming

## Abstract

Genome-scale metabolic models (GEMs) are increasingly applied to investigate the physiology not only of simple prokaryotes, but also eukaryotes, such as plants, characterized with compartmentalized cells of multiple types. While genome-scale models aim at including the entirety of known metabolic reactions, mounting evidence has indicated that only a subset of these reactions is active in a given context, including: developmental stage, cell type, or environment. As a result, several methods have been proposed to reconstruct context-specific models from existing genome-scale models by integrating various types of high-throughput data. Here we present a mathematical framework that puts all existing methods under one umbrella and provides the means to better understand their functioning, highlight similarities and differences, and to help users in selecting a most suitable method for an application.

## Introduction

Genome-scale metabolic models (GEMs) have become a useful tool to investigate metabolism. They present numerous applications, from basic research on metabolic functioning and cell physiology (Bordbar et al., 2014) to the design of novel strains for improving biotechnological processes to the analysis of metabolic diseases and the quest for novel drug targets (Milne et al., 2009; Garcia-Albornoz and Nielsen, 2013; Agren et al., 2014). Although historically biased toward microorganisms, a number of GEMs have recently been reconstructed for several plant species, including: *Arabidopsis thaliana* (Poolman et al., 2009; De Oliveira Dal'Molin et al., 2010; Saha et al., 2011; Arnold and Nikoloski, 2014), maize (Saha et al., 2011), maize and other C4 plants (Dal'Molin et al., 2010), rice (Dharmawardhana et al., 2013; Poolman et al., 2013) and algae (Chang et al., 2011; Gomes de Oliveira Dal'Molin et al., 2011). This late development of plant GEMs is largely due to the particular challenges of modeling plant metabolism, (in general more complex and characterized by cellular compartmentalization and an extensive secondary metabolism) and a lower coverage of annotated metabolic genes in plants in comparison with, much simpler and more experimentally accessible, microorganisms. The development plant GEMs and particular challenges are summarized in De Oliveira Dal'Molin and Nielsen (2013) and Sweetlove and Ratcliffe (2011).

The success of GEMs is largely due to their integrative nature, representing the whole known network of biochemical reactions of a given organism, and the possibility to readily use them in a mathematical model. This mathematical model can be further interrogated with powerful methods from constraint-based analysis (Lewis et al., 2012), whereby a system of mass balance equations at steady state, with additional thermodynamic and capacity constraints, define a solution space of feasible metabolic flux values. The imposed constraints may also lead to inconsistencies in the original metabolic model; for instance, by enforcing blocked reactions, i.e., reactions incapable of carrying nonzero flux at steady state. Flux balance analysis (Orth et al., 2010) represents a prominent method within constraint-based analysis, and has been widely applied to explore cell physiology. It assumes that cells adapt metabolic fluxes to optimize a certain objective function (i.e., a linear combination of metabolic fluxes). Although GEMs and constraint-based methods are convenient when modeling the entirety of known metabolism, mainly due to the smaller number of parameters to be measured (e.g., external fluxes), other available methods, such as stochastic (Wilkinson, 2009; Ullah and Wolkenhauer, 2010) or deterministic (Link et al., 2014), kinetic models may offer an alternative strategy, particularly for modeling smaller cellular subsystems. The latter is particularly the case when the focus is modeling of the dynamics of metabolite concentrations and/or of regulatory mechanisms. However, due to the dependence on a large number of (not readily measurable) parameters and the computational demand, these methods usually are not scalable. Interestingly, some hybrid approaches have been proposed merging constraint-based and kinetic methods, which may overcome individual limitations of both methods, ultimately resulting in better predictions (Jamshidi and Palsson, 2010; Soh et al., 2012; Chakrabarti et al., 2013; Chowdhury et al., 2014).

The recent advent of high-throughput technologies has propelled the GEM community to develop new methods for integrating high-throughput data into existing metabolic models. In general, these methods employ data to (1) improve flux predictions through further constraining of the solution space (Colijn et al., 2009; Chandrasekaran and Price, 2010; Jensen and Papin, 2011; Collins et al., 2012; Lee et al., 2012), and/or (2) extract context-specific metabolic models, which are a subset of the original GEM (Becker and Palsson, 2008; Shlomi et al., 2008; Jerby et al., 2010; Agren et al., 2012; Wang et al., 2012; Schmidt et al., 2013; Vlassis et al., 2014). In the first case, the metabolic model serves as a scaffold to analyze complex data sets from different sources, e.g., transcript, protein or metabolite profiles. The second case is motivated by the mounting evidence suggesting that the structure of a given metabolic network changes across different conditions, e.g., environmental changes, developmental stages as well as different cell-types or tissues. Therefore, in context-specific metabolic models only a subset of the reactions from the original GEM carry flux, and are considered active. This is of particular importance when tackling multicellular organisms, like plants, where multiple cell types with specialized metabolic functions coexist and cooperate. Following this line, a number of tissue-specific models have been reconstructed in Mintz-Oron et al. (2012) using one of such methods (the MBA, discussed below) together with a genome-scale model of *Arabidopsis* and publicly available tissue-specific expression profiles. However, other, manual, approaches have been used to take into account cell and tissue type in plant GEMs; for instance, in C4GEM, two cell types are modeled: bundle sheath and mesophyll cells, to capture the typical C4 carbon fixation physiology (Dal'Molin et al., 2010). In Grafahrend-Belau et al. (2013) authors go further in scope to model the metabolism of a whole barley plant, using four organ-specific models (leaf, stem, seed, and root) that are interconnected through two exchange compartments (the phloem and external environment). Here, we will use the generic term *context* for any of the particular conditions that may occur.

High-throughput data sets can be divided in hierarchical categories that correspond to different cellular processes. On one hand, transcript profiles capture the instantaneous expression state of a given genome under a particular condition. They have the greatest coverage, since usually all known genes are considered. They are also the most accessible in terms of experimental tractability, due to the availability of classical technologies (e.g., microarray) as well as modern developments (i.e., RNAseq). However, gene expression is also at the top of the hierarchical chain of events that govern metabolic fluxes, which may explain the relatively low correlation values between these two quantities, as reported in previous works (Yang et al., 2002; Rossell et al., 2006; Daran-Lapujade et al., 2007; Moxley et al., 2009). Protein levels may be more concordant to metabolic fluxes, and hence several methods have aimed to incorporate this source of evidence (Jerby et al., 2010; Agren et al., 2012, 2014; Bordbar et al., 2012). However, existing measurement techniques, mainly based on the combination of chromatography and mass spectrometry (Schulze and Usadel, 2010), do not offer an extensive coverage of the proteome. Finally, metabolites directly relate to metabolic fluxes, since they play the role of substrates and products of metabolic reactions. Therefore, metabolite levels may better reflect the actual state of a metabolic network. Unfortunately, current measurement methods do not permit full coverage of the metabolome to describe the metabolic state of the entire network (Fernie, 2007). Despite this shortcoming, integration of metabolite levels can substantially improve flux predictions or the extraction of context-specific models, especially when they are combined with protein and/or gene expression levels (Yizhak et al., 2010; Kleessen et al., 2012).

Several recent comprehensive reviews provide extensive coverage of computational methods for integrating high-throughput data in GEMs (Joyce and Palsson, 2006; Blazier and Papin, 2012; Lewis et al., 2012; Hyduke et al., 2013), with a recent study offering a critical systematic evaluation and performance comparison (Machado and Herrgård, 2014). Here we propose a mathematical framework that groups existing methods for context-specific model extraction in three families. This framework provides not only a mere classification but also the means to better understand the rationale behind methods and highlight their common principles and differences. We also propose a flowchart to guide interested users in selecting a method to apply in a particular setting. In the following, for each family of methods, we present its general functioning and mathematical objective, discuss its advantages and disadvantages, and we also highlight particularities of each method.

## Generalization of methods for extraction of context-specific models

**Figure 1:**
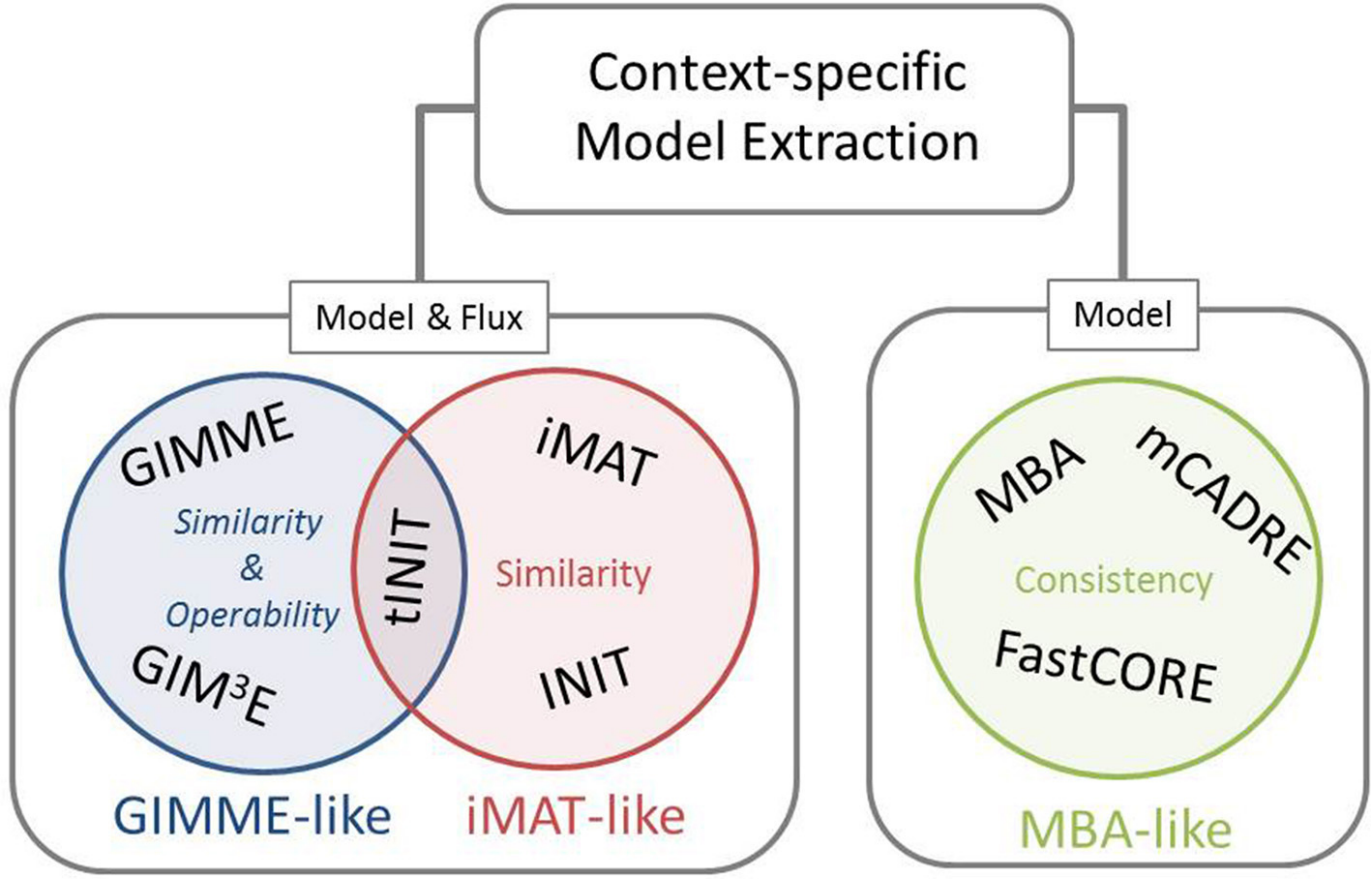
**Existing methods for context-specific model extraction can be classified in three families: GIMME-, iMAT-, and MBA-like**. This classification is based on the mathematical objective followed by the methods, i.e., similarity and operability, similarity or consistency. Moreover, methods can perform a model extraction and a flux prediction (GIMME- and iMAT-like families) or only a model extraction (MBA-like family). Although similar in formulation to INIT, the metabolic task constraint imposed by tINIT renders this algorithm close to the objective of the GIMME-like family (see main text).

Our framework for classification of the existing methods for extraction of context-specific models simultaneously offers a generalization of the mathematical and algorithmic formulation. With respect to the employed objective, these methods can be divided into three main families, namely: GIMME-, iMAT-, and MBA-like families, termed after the first representative method in each class (Figure 1). The objective employed by the GIMME-like family corresponds to the similarity of the flux phenotype to data, which is to be maximized while guaranteeing a given Required Metabolic Functionality (RMF), such as: growth or ATP production. In contrast, the iMAT-like family of methods aims at maximizing the similarity of the flux phenotype to data without imposing any RMF. Finally, the MBA-like family uses model consistency as objective, which refers to a final context-specific model without any blocked reaction. The mathematical generalization of each family of methods captures these principles, highlights the similarities, and serves as a scaffold to frame particularities of each method.

### GIMME-like family

The GIMME-like family encompasses the GIMME method (Becker and Palsson, 2008) and GIM^3^E, as an extension (Schmidt et al., 2013). This family reconstructs a context-specific model in two steps: First, it optimizes an objective function, the RMF, by using the classical linear programming (LP) formulation of flux balance analysis which imposes mass balance and thermodynamic constraints. This objective function is assumed to be the main cellular task in the investigated condition. It then involves solving a second LP that minimizes a penalty function, corresponding to the discrepancies between flux values and the respective transcript levels, with the additional constraint that the flux through the previous RMF must be above a given lower bound (e.g., a fraction of the optimum value found by flux balance analysis). The methods included in this family mainly differ in the way the discrepancies are minimized in the second step, the type of high-throughput data used, and in the treatment of reversible reactions, as detailed below. Box 1 displays the formulations and the generalization of this family (consult Table 1 for a glossary of used symbols).

Box 1 **Mathematical formulations of the GIMME-like family: (A) generalization of the family, (B) GIMME and (C) GIM^3^E. In (B,C) only the second LP is represented (see main text). In (C) individual reaction-associated gene penalties are first calculated and then mapped to the corresponding reaction to obtain the reaction penalty, here represented by the function: *gpr*(*I_max_−I_d_ i*) (see main text). Consult the Glossary of symbols for notation.**

**Table:** 

**(A) GIMME-like family**	**(B) GIMME**	**(C) GIM^3^E**
1. function *GIMME-like* (S,RMF,k)	$\underset{v}{\min}\sum_{i\;\in \;\{i:d_{i}\;<\;c\}}\left(d_{i}-c\right)*v_{i}$	$\underset{v}{\min}\sum_{i\;\in \;R_{G}}gpr\left(I_{max}-I_{d}\;i\right)*v_{i}$
2. max_v_ RMF		
*s.t.*	*s.t.*	*s.t.*
SV = 0		
v_min_ ≤ v ≤ v_max_	SV = 0	SV = 0
3. min_v_ IS	*v_min_ ≤ v ≤ v_max_*	*v_min_ ≤ v ≤ v_max_*
*s.t.*		
SV = 0	*RMF* = k * RMF	*RMF* = k * RMF
v_min_ ≤ *v* ≤ *v*_max_		*v_i_ ≥ ∈, i ∈ KeyMetSink*
RMF = k^*^RMF_opt_		*v*_*i*_ = *y* * *v*_*for*(*i*)_ − (1 − *y*) * *v*_*rev*(*i*)_, *i* ∈ *Rev*
k ∈ [0, 1]		
4. end function		*y* ∈ {0, 1}

**Table 1:** **Glossary of symbols**.

**Symbol**	**Definition**
*R_G_*	Set of reactions of the generic model
*R_P_*	Set of reactions of the (partial) context-specific model
*C*	Core set of reactions
*C_H_*	Core set of reactions with high likelihood
*C_M_*	Core set of reactions with moderate likelihood
*N_C_*	Non-core set of reactions
*R_Nc_*	Subset of reactions from N_*C*_
KeyMet	Set of key metabolites (holding positive evidence)
KeyMetProd	Set of reactions producing a key metabolite (with positive evidence)
KeyMetSink	Set of sink reactions for a metabolite with positive evidence
MetTask	Set of reactions participating in a given metabolic task (a linear combination of a subset of the generic model)
Negative	Set of reactions whose associated transcript/s hold/s negative evidence (non-expressed in any condition)
Rev	Set of reversible reactions of the generic model
*R_H_*	Set of reactions with high associated expression value
*R_L_*	Set of reactions with low associated expression value
*K*	Weighting factor (scalar), typically k∈[0, 1]
*C*	User-defined threshold for expression values
ϵ, δ	User-defined small positive value
*W*	Vector of weighting factors (arbitrary function of experimental evidence)
*S*	Stoichiometric matrix
*V*	Vector of flux values
*v_max_, v_min_*	Boundary conditions for V (physiologically maximal and minimal flux capacity)
*v_for_, v_rev_*	Forward and reverse senses of reversible reactions
*B*	Vector of concentration rates
*D*	Vector of data values
IS	Inconsistency score
FVA	Flux Variability Analysis
RMF, RMF_*opt*_	Required Metabolic Functionality, RMF optimum value as calculated by FBA
*I_max_, I_d_*	Gene expression measured intensities, maximum gene intensity (for a given sample) and intensity value for a particular gene, respectively

#### GIMME

In GIMME, the penalty function is termed *inconsistency score*. This function penalizes flux values of reactions whose associated expression levels are below a user-defined cut-off (i.e., threshold). More specifically, the inconsistency score is given by the dot product of the flux distribution and the reaction penalty, defined as the vector difference of the associated expression values from the threshold. The reaction associated expression level is obtained following the standard GPR rules (Becker and Palsson, 2008), which take into account the presence of isoenzymes and protein complexes. Although transcript profiles were used in the original formulation, a variant called GIMMEp allows for the integration of proteomic data (Bordbar et al., 2012). The result of applying this algorithm is a flux distribution which ensures that a given RMF can be carried out and is as consistent as possible to the employed data.

#### GIM^3^E

GIM^3^E introduces several modifications to the original GIMME. First, it allows integration of metabolomics data, imposing a nonzero flux value to reactions involving a metabolite for which there is evidence of being synthesized in an investigated condition. Second, it modifies the definition of the reaction penalty; here, the penalties for all reaction-associated genes are determined separately and are then mapped to the reaction following the GPR rules. Moreover, the penalties are calculated as the distance between each transcript and the maximum expression level of the set. Consequently, after mapping transcript penalties all reactions obtain a penalty value, rather than only the set below the threshold which is the case in GIMME. Finally, GIM^3^E takes into account directionality of reversible reactions by constraining them to operate in only one direction, which is modeled by introducing a binary variable for the direction of choice. As a result, GIM^3^E is formulated as a mixed integer linear program (MILP), which is more computationally challenging than the LP formulation of GIMME.

#### Advantages and disadvantages of the GIMME-like family

When a given RMF operates in different contexts, the operability constraint may lead to more accurate context-specific model reconstructions and flux distributions. This issue has been evaluated in a recent review (Machado and Herrgård, 2014), demonstrating that methods which do not impose network operability were incapable of predicting growth using a yeast metabolic model. Furthermore, the total sum of the inconsistency score also quantifies the correspondence of the RMF to the set of expression data, which may provide further insights into cellular functionality.

Nevertheless, while the selection of a RMF can be a relatively easy task for prokaryotes, whereby experimental evidence supports the choice of cellular growth or biomass maximization as a plausible RMF, this task is much more challenging for eukaryotic organisms, especially the multicellular. In this case, choosing a RMF for a given tissue or cellular type is a complicated task, as each cell type is specialized in certain biochemical functions, modulated on the level of the entire organism. Therefore, methods that do not require a RMF may be applied easier to models of multicellular organisms.

There are existing implementations for both methods: GIMME can be executed using the c*reateTissueSpecificModel* function built in the COBRA toolbox within the Matlab environment (Schellenberger et al., 2011). The GIM^3^E implementation is however built under Python (“The OpenCOBRA Project^1^,” n.d.).

### iMAT-like family

The iMAT-like family comprises three methods, iMAT (Shlomi et al., 2008), INIT (Agren et al., 2012) and its extension, tINIT (Agren et al., 2014), which also aim at extracting a context-specific model compatible with a given data set. However, in contrast to the GIMME-like family of methods, the iMAT-like family does not assume a RMF achieved by the cell. More specifically, these methods maximize the number of matches between reaction states (i.e., active or inactive) and corresponding data states (i.e., expressed or not non-expressed). The mathematical formulation results in a MILP, in which the value of the binary variable denotes the most concordant reaction state for a given (data) context. Although sharing the general strategy, iMAT, INIT and tINIT differ considerably respecting to how they deal with data: iMAT integrates data in the constraints, INIT and tINIT do so directly in the objective function. See Box 2 for mathematical formulations and generalization of the family, and Table 1 for a glossary of used symbols.

Box 2 **Mathematical formulations of the iMAT-like family: (A) generalization of the family, (B) iMAT, and (C) INIT. In (C), the tINIT extension is displayed in blue.. Consult the Glossary of symbols for notation.**

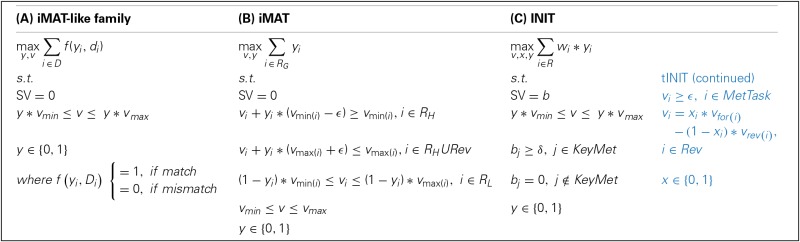

#### iMAT

The algorithm first classifies reactions into two groups based on a previously defined threshold for the corresponding expression data; this results in the groups of reactions with a high and low associated expression values. It then maximizes the number of matches between a reaction state, defined through a minimum flux value, and the group to which the reaction belongs. Thus, if a reaction is included in the highly expressed group, the aim is to obtain a flux value over the minimum, which is performed by solving the MILP in Box 2.

Several network states can yield the same overall similarity to expression data, i.e., multiple flux distributions may yield the same objective function value. iMAT tackles this issue through an adapted flux variability analysis (FVA): First, it forces each reaction to be active and evaluates the similarity, and then repeats the process in a similar way by forcing each reaction to be inactive. The final outcome is computed by comparing the two obtained similarities. A reaction is termed active if its inclusion results in higher similarity to data, and it is termed as inactive, if its inclusion decreases this similarity. In the case that both similarities are equal, iMAT categorizes the reaction as undetermined.

#### INIT

INIT was optimized to integrate evidences from the Human Protein Atlas, although expression data are integrated when proteomic evidences are missing. In this case, INIT does not group reactions in categories in contrast to iMAT. Instead, it adopts experimental data to weight the binary variable of the corresponding reaction, whereby the weight is a function of experimental data (e.g., gene expression profiles) or a set of arbitrary numbers that quantify the color code of the entries of the Human Protein Atlas. In addition, INIT imposes a positive net production of metabolites for which there is experimental support for that context or tissue. Hence, when a metabolite is experimentally determined to be present, its net production is forced to comply with a given lower bound. As a result, INIT allows the integration of metabolomics data in a qualitative way. This method has been applied to generate a human metabolic reaction database (“Human Metabolic Atlas^2^,” n.d.) where several tissue-specific model reconstructions can be examined.

#### tINIT

tINIT, an extension of INIT, has been recently proposed (Agren et al., 2014). Here, the main innovation comes with the definition of a set of metabolic tasks that the final context-specific model must perform. These tasks can represent production or consumption of a certain metabolite or the activation of entire pathways that are known to occur in a given context. Furthermore, reversible reactions are constrained to operate in only one direction, which introduces an extra binary variable. The user can choose between establishing a net production of certain metabolites, as in INIT, or maintaining the steady state. Finally, the task-driven strategy of tINIT renders this algorithm close to the principles of the GIMME-like family, since it aims to obtain operational context-specific models in coherence with experimental data.

#### Advantages and disadvantages of the iMAT-like family

The main advantage of this family of methods is the independence of a RMF; therefore, these methods are convenient for extracting context-specific models when no specific RMF is known to dominate the context, which is often the case for tissue-specific models of multicellular organisms. However, MILP problems are computationally more challenging in comparison to LP problems, and may, in general, require longer computation time. This is particularly the case of iMAT, in which two MILPs have to be solved in the modified FVA per reaction. iMAT can be easily implemented using the c*reateTissueSpecificModel* function of the COBRA toolbox, although only one MILP is solved in this implementation, ultimately reducing the computation time at the expense of neglecting the exhaustive search through the space of possible multiple optima. The INIT and tINIT methods are integrated within the RAVEN toolbox (Agren et al., 2013) for Matlab, and the user can define a set of metabolic tasks to be performed (tINIT) or run the algorithm without any (INIT). Note that selection of direction in reversible reactions is disabled by default.

### MBA-like family

The MBA-like family is composed of MBA (Jerby et al., 2010), mCADRE (Wang et al., 2012) and FastCORE (Vlassis et al., 2014). While previous methods perform both a flux prediction and a context-specific model reconstruction, MBA-like methods only return a context-specific model as output. This family *a priori* categorizes reactions in two sets, the core and the non-core. The core set includes those reactions with positive evidences (e.g., high-throughput data and/or well-curated biochemical knowledge) of being active in a certain context. Once these sets are defined, the MBA-like methods prune the GEM by eliminating non-core reactions that are unnecessary to ensure consistency in the core set, i.e., no blocked reaction is allowed in the final model. Thereby, all reactions must carry non-zero flux in at least one feasible solution. As a result, checking model consistency is a crucial part of these methods and also the main difference in comparison to the other methods. FVA have been used to pinpoint blocked reactions, but it is computationally expensive since it requires solving two optimization problems per reaction (Mahadevan and Schilling, 2003). Thus, the major changes in formulation are due to finding faster alternatives to perform the same task. However, other differences arise when defining the core set and during the pruning process. Box 3 shows the three MBA-like algorithms in pseudocode, as well as the generalization of the family (consult Table 1 for a glossary of used symbols).

Box 3 **Pseudocode describing algorithms of the MBA-like family corresponding to: (A) the generalization of the family, (B) MBA, (C) FastCORE, (D) mCADRE. The *CheckModelConsistency* function (E) of MBA and *FindSparseMode* (F) of FastCORE are presented separately. Consult the Glossary of symbols for notation.**

**Table:** 

**(A) MBA-like family**	**(B) MBA**	**(C) FastCORE**
function *MBA-like* (R_G_,C) R_P_ ← C N_C_ ← R_G_\C blockedReactions ← *CheckModelConsistency*(R_P_) if blockedReactions = ∅ return R_P_ end if while blockedReactions ≠ ∅ R_P_ ← R_P_ ∪ R_Nc_ N_C_ ← N_C_\R_Nc_ blockedReactions←*CheckModelConsistency*(R_P_) end while return R_P_ end function	function *MBA* (R_G_,C_H_,C_M_) R_P_ ← R_G_ N_C_ ← R_G_\(C_H_∪C_M_) choose random permutation, P, from N_C_ for each reaction r ∈ P, R_P_ ← R_P_\r blockedReactions ← *CheckModelConsistency*(R_P_) e_H_ ← blockedReactions∩C_H_ e_M_ ← blockedReactions∩C_M_ e_Nc_ ← blockedReactions\(C_H_∪C_M_) if (|e_H_| = 0) AND (|e_M_| < k^*^|e_Nc_|), R_P_ ← R_P_\(e_M_∪e_Nc_) end if end for end function	function *FastCORE*(R_G_,C) R_P_ ← ∅ J ← C P ← R_G_\C while J ≠ ∅ R_P_ ← R_P_∪*FindSparseMode*(J,P) J ← J\R_P_ P ← P\R_P_ end while end function
**(D) mCADRE**	**(E) CheckModelConsistency (MBA)**	**(F) FindSparseMode (FastCORE)**
function *mCADRE* (R_G_,C) R_P_ ← R_G_ N_C_ ← R_G_\C for each reaction r∈N_C_, R_P_ ← R_P_\r blockedReactions ← *CheckModelConsistency*(R_P_) e_C_ ← blockedReactions∩C e_Met_ ← blockedReactions∩KeyMetProd e_NC_ ← blockedReactions∩N_C_ if r∉Negative, if (|e_C_| = 0) AND (|e_Met_| = 0), R_P_ ← R_P_\r∪e_Nc_ end if else if r ∈ Negative, if (|e_Met_| =0) AND (|e_C_| < k*|e_Nc_|), R_P_ ← R_P_\r∪e_Nc_∪e_C_ end if end if end for end function	function *CheckModelConsistency* (R_P_) max_*v*_ ∑_i ∈ R_P__ *v_i_* *s.t.* SV = 0 v_min_ ≤ v ≤ v_max_ R_P_ ← R_P_\{i ∈ R_P_: v_i_ ≥ ε} min_*v*_ ∑_*i* ∈ *R_P_* ∩ *Rev*_ *v*_*i*_ *s.t.* SV = 0 v_min_ ≤ v ≤ v_max_ R_P_ ← R_P_\{i ∈ R_P_: v_i_ ≥ ε if {i∈R_P_: v_i_ ≥ ε} = ∅, select random reaction, i, and solve FVA R_P_ ← R_P_\{i : |v_i_| ≥ ε} end if end function	function *FindSparseMode* (J,P) max_*v,z*_ ∑_*i* ∈ *J*_ *z*_*i*_ *s.t.* z_i_ ∈ [0,ε], ∀i ∈ J, z_i_ ∈ ℝ_+_ v_i_ ≥ z_i_, ∀i∈J SV = 0 v_min_ ≤ v ≤= v_max_ K ← {i∈J:v_i_ ≥ ε} min_*v,z*_ ∑_*i* ∈ *P*_ *v*_*i*_ *s.t.* v_*i*_ ∈[−z_i_,z_i_], ∀i∈P, z_i_ ∈ ℝ_+_ v_i_ ≥ ε, ∀i∈K SV = 0 v_min_ ≤ v ≤ v_max_ end function

#### MBA

MBA divides the core set in two subcores: a set with high likelihood to be present in the context-specific model (*C_H_*), if evidence comes from well-curated biochemical knowledge in that particular context, and a set with moderate likelihood (*C_M_*) if evidence comes from context-specific high-throughput data. The algorithm performs the pruning iteratively and randomly by selecting a non-core (*N_C_*) reaction to be eliminated, and checking consistency at each step: if *C_H_* and a user-defined fraction of *C_M_* remain unblocked, MBA removes the reaction out of the model along with *C_M_* and *N_C_* corresponding blocked reactions. This routine is repeated until no reaction is left in *N_C_*. The topology of the final model clearly depends on the order in which non-core reactions are eliminated. Therefore, to remove artifacts due to the order, the algorithm is repeated a number of times (1000 in Jerby et al., 2010) to obtain a population of context-specific models. Later, reactions are ranked according to their occurrence in the population and added up to C_*H*_ until a consistent model is obtained. MBA proposes an alternative to FVA to check consistency in a more efficient way: First, it solves a LP problem which maximizes the total sum of fluxes. It then removes active reactions (i.e., carrying non-zero flux) and repeats the LP over the remaining set of reactions. If no reaction is found to be active, FVA is applied to each reaction to determine whether it is blocked. The process is repeated until all reactions have been classified either as blocked or unblocked.

#### mCADRE

A prominent characteristic of mCADRE lies in ranking reactions of the genome-scale reconstruction according to three scores: expression-, connectivity-, and confidence-level-based. In addition, this ranking determines the core set of reactions as well as the order by which non-core reactions are eliminated. The core is determined by fixing a threshold value to the expression-based score; therefore, reactions whose values are above the threshold are included in the core, and the rest constitute the non-core reactions. Unlike other methods, the expression-based score does not directly consider the levels of expression. Instead, it calculates the frequency of expressed states over a battery of transcript profiles in the same context, and, thus, requires a previous binarization of the expression data. Reactions outside the core are then ranked according to the connectivity-based score, which assesses the connectedness of adjacent reactions, and the confidence level-based score, which accounts for the type of evidences supporting a reaction in the genome-scale reconstruction.

Non-core reactions are in turn sequentially removed according to the previous ranking, and consistency is evaluated. Here, mCADRE presents two other innovations: it defines a set of key metabolites, with positive evidences of appearing in the context-specific model reconstruction, and relaxes the stringent condition of including all core reactions in the final model. More specifically, a reaction can only be eliminated if it does not prevent the production of a key metabolite and if it is unnecessary to ensure core consistency. However, if evidence exists for the respective transcript to be unexpressed in any of the context-specific samples, mCADRE allows the elimination of the reaction even if it blocks some of the core reactions. To this end, two conditions have to be satisfied: (1) production of key metabolites is not impaired and (2) the relation between the number of blocked core and non-core reactions matches a predefined ratio. To check model consistency, mCADRE maintains the procedure proposed in MBA, although adapted to use FastFVA (Gudmundsson and Thiele, 2010) instead of maximizing the total sum of flux values. mCADRE has been used to create the Tissue-Specific Encyclopedia of Metabolism (“Tissue-Specific Encyclopedia of Metabolism^3^,” n.d.) using the Recon1 human metabolic reconstruction (Duarte et al., 2007) and data from the Gene Expression Barcode Project (McCall et al., 2014) to extract 126 tissue-specific model reconstructions.

#### FastCORE

While FastCORE aims also at obtaining a minimal consistent model containing all core reactions, typical for this family of methods, it differs principally from MBA and mCADRE in the algorithmic strategy. Instead of eliminating one non-core reaction followed by consistency evaluation at each step, FastCORE solves two LPs: The first LP maximizes the cardinality of the core set of reactions, computed as the number of reaction values above a small positive constant. On the other hand, the second LP minimizes the cardinality outside the core set by minimizing the *L*_1_-norm of the flux vector, under the constraint that the entire core set must remain active. These two LPs are repeatedly applied in alternating fashion until the core set is consistent, whereby activation of all core reactions is ensured while including a minimum set of non-core reactions in the final model. To deal with reversible reactions, FastCORE evaluates both directions by changing the sign of the corresponding column of the stoichiometric matrix.

#### Advantages and disadvantages of MBA-like methods

One of the main advantages of this family over other methods is the possibility to integrate multiple data sets of different nature together with well-curated biochemical knowledge. Defining a core set of reactions from such a diverse collection of experimental evidence may increase the confidence for a particular set of reactions to appear in a certain context (e.g., tissue), as missing information on one data set can be complemented by another. Moreover, imposing the whole core set inclusion can be highly advantageous, as reactions with overwhelming evidence would always be included in the context-specific model. Moreover, like the iMAT-like family, MBA-like methods are independent of a RMF and, hence, appropriate to be employed if no RMF is known to operate in a given context. Nevertheless, we would like to emphasize that MBA-like methods provide only a context-specific model reconstruction, in contrast to the iMAT-like methods which generate both a context-specific reconstruction and a flux distribution.

MBA-like methods follow two ways to define the core set of reactions: MBA takes into account well-curated biochemical knowledge and a variety of experimental data (e.g., transcript, protein, metabolite, and/or metabolic flux profiles). While this approach to define the core set of reactions may be more accurate, it is also time-consuming due its manual nature. On the other hand, the definition of the core set in mCADRE allows for full automation, since it relies only on determining a threshold to expression-based evidence.

In terms of computation time, FastCORE outperforms the contending alternatives. Therefore, it has advantages over other methods when computing time is the limiting resource, provided that a properly defined core set is given (note that FastCORE does not provide an operational definition of a core set). The good time-related performance of FastCORE is due to two main innovations: First, the maximization of the cardinality represents a softer objective than the maximization of the total sum of flux values (used in MBA), since fluxes are only required to be above a small positive value. Consequently, solving this optimization problem usually results in more active reactions per iteration than the MBA counterpart. Second, the computation of the *L*_1_-norm to prune non-core reactions renders the pruning step more efficient due to the possibility to remove a once more than one reaction. These modifications make FastCORE the fastest algorithm in this family of methods, as it is able to extract a context-specific model in a computational time two to three orders of magnitude smaller than that expended by mCADRE and MBA (Vlassis et al., 2014). Finally, both mCADRE and FastCORE can be run under the Matlab environment (“FastCORE in COBRA toolbox^4^,” n.d., “mCADRE source code^5^,” n.d.).

## Conclusions

Here we presented a classification of the existing approaches for extracting context-specific metabolic models. We classified the methods into three families according to their mathematical formulation. Furthermore, we also proposed a mathematical generalization for each family, which summarizes the fundamental principles shared by its members.

Altogether, the classification and generalization constitutes a mathematical framework that aims to fulfill three main purposes: First, it provides a better understanding of the rationale behind methods, allowing an easy inspection of its main characteristics as well as highlighting the advantages and shortcomings. Second, such structured knowledge may facilitate the envisioning of novel approaches to extract context-specific models. Third, it may help users in choosing a best suited method for their particular problem, since the classification outlines the differences in the data and knowledge requirements as input to the particular methods.

**Figure 2:**
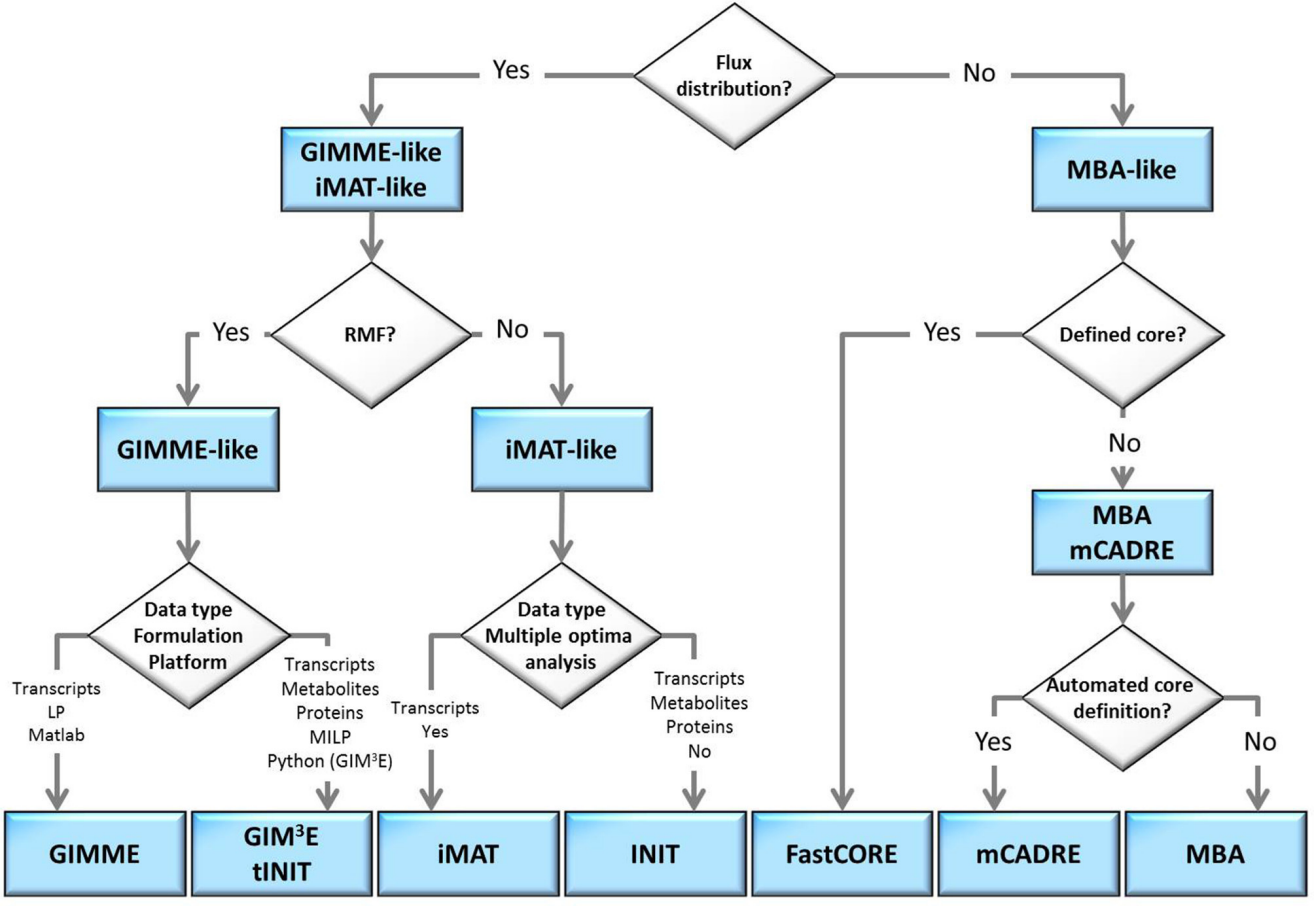
**Optimal choice of methodologies when tackling a context-specific reconstruction problem**. The choice can be made by answering a few questions, in a flowchart manner, related to: demand of model extraction and flux prediction, knowledge on a required metabolic functionality, the type of experimental data available or the computational platform.

The flow-chart on Figure 2 demonstrates that an optimal choice with respect to the parameters and available data (Table 2) may be executed in a simple and concise manner by answering few questions. Initially, one may select between methods that perform both, a model extraction and a flux prediction (GIMME-and iMAT-like families), or methods which only provide a context-specific model (MBA-like families). To further select between the GIMME- and the iMAT-like families, one can take into account if a RMF is known to operate in the context under consideration. In that case the GIMME-like family may provide the method of choice, since the resulting model would be guaranteed to include the RMF. Selection of GIMME or GIM^3^E may depend on the interest to integrate metabolomics data along with transcripts profiles, the computational platform of the current implementations, or the difference in computing time. For instance, the choice is between the COBRA toolbox in Matlab, for GIMME, or its version in Python, for GIM^3^E (“The OpenCOBRA Project,” n.d.), or between the LP formulation of GIMME, vs. the more computationally demanding MILP of GIM^3^E.

**Table 2:** **Summary of methods for context-specific metabolic model extraction**.

	**Parameters**	**Formulation**	**Implementation**	**Omics data**	**RMF**	**Flux distribution**
GIMME	*c, k, v*_max_, *v*_min_	LP	COBRA (Matlab)	Transcripts	Required	Yes
GIM^3^E	*k, v*_max_, *v*_min_	MILP	COBRA (Python)	Transcripts, metabolites	Required	Yes
iMAT	Data discretization^*^, *v*_max_, *v*_min_	⟳ MILP	COBRA (Matlab)	Transcripts, proteins	Unrequired	Yes
INIT/tINIT	Data discretization^*^, ϵ, δ, *v*_max_, *v*_min_	MILP	RAVEN (Matlab)	Transcripts, proteins, metabolites	Optional	Yes
MBA	Data discretization^*^, *k*, ϵ, *v*_max_, *v*_min_	⟳ LP	-	Curated biochemical knowledge, transcripts, proteins, metabolites, fluxes	Unrequired	No
mCADRE	Data discretization^*^, *k*, ϵ, *v*_max_, *v*_min_	⟳ LP	Matlab	Transcripts, metabolites	Unrequired	No
FastCORE	ϵ, *v*_max_, *v*_min_	⟳ LP	COBRA(Matlab)	-	Unrequired	No

* *These methods discretize data following a heuristic approach without any concrete parameter. ⟳ stands for iteratively repeated*.

Without the information about the operability of a particular RMF in a given context, the iMAT-like family may provide the method of choice. To select between iMAT and INIT one could take into account the flexibility on integrating different types of experimental data, since iMAT was developed to integrate transcript profiles, whereas INIT can integrate semi-quantitative proteomic data, transcript profiles and metabolic evidences. In addition, one could consider the possibility of the method to discriminate between multiple optima with same similarity score, together with the computational cost for performing this task.

In contrast, if only a context-specific model extraction is required, one may opt for any of the presented method. However, the methods in the MBA-like family have some advantageous properties, namely, the integration of a variety of experimental data sources and the inclusion of reactions for which there is strong experimental evidence in the context-specific reconstruction. One may then choose based on the core set definition of each method as well as on the total computational time required. The MBA-like family proposes two ways to define the core: the MBA semi-automated procedure, whereby reactions are included in the core set if there is sufficient positive evidence across different databases, and the mCADRE automated procedure, whereby reactions are included if the expression value of the respective transcript is larger than a given threshold. Thus, if an appropriate number of databases contain experiments about the context of interest and the computation time is not a primary limitation, the MBA core definition may be a suitable alternative. As previously commented, this procedure can cross-validate the confidence on a reaction to belong to a certain context, due to the simultaneous usage of several databases. Subsequently, one can readily employ MBA to extract the context-specific model, or can opt for FastCORE, which can perform the extraction, using the previously defined core, in a more efficient way. On the other hand, mCADRE could be preferentially applied when an automated core definition is preferred. Moreover, the mCADRE relaxation of whole core inclusion can improve accuracy when a core reaction diminishes the overall coherence with respect to the data, through the inclusion of non-core reactions with negative evidences to ensure consistency. Finally, one can also apply FastCORE to a core set defined in an automated way to benefit of its rapid computation. However, neglecting the characteristic core relaxation and ranking of non-core reactions of mCADRE.

Development of new approaches for extraction of context-specific metabolic models can further expand on the advantages of the existing methods, while facilitating efficient computation accounting for the shortcomings. This will allow rapid devising of context-specific models and their interconnection in larger multilevel models, typical for complex eukaryotes, to allow for more realistic simulation scenarios.

### Conflict of interest statement

The authors declare that the research was conducted in the absence of any commercial or financial relationships that could be construed as a potential conflict of interest.

## Footnotes

^1^Retrieved from http://opencobra.sourceforge.net/openCOBRA/Welcome.html

^2^Retrieved from http://www.metabolicatlas.com/

^3^Retrieved from https://price.systemsbiology.net/tissue-specific-encyclopedia-metabolism-tsem

^4^Retrieved from https://github.com/opencobra/cobratoolbox/tree/cd4368bda07ef4d63486a3683865f4d9f3bc53fe

^5^Retrieved from https://price.systemsbiology.net/tissue-specific-encyclopedia-metabolism-tsem
